# Piperonal synthase from black pepper (*Piper nigrum*) synthesizes a phenolic aroma compound, piperonal, as a CoA-independent catalysis

**DOI:** 10.1186/s13765-022-00691-0

**Published:** 2022-03-24

**Authors:** Zhehao Jin, Dae-Kyun Ro, Soo-Un Kim, Moonhyuk Kwon

**Affiliations:** 1 Research Institute of Agriculture and Life Sciences, Seoul National University, 1 Gwanak-ro, Gwanak-gu, Seoul, 08826 Republic of Korea; 2 Present Address: Institute of Synthetic Biology, Shenzhen Institutes of Advanced Technology, Chinese Academy of Sciences, Shenzhen, 518055 Guangzhou China; 3 Department of Biological Sciences, University of Calgary, Calgary, AB T2N 1N4 Canada; 4 Division of Applied Life Science (BK21 Four), ABC-RLRC, PMBBRC, Gyeongsang National University, Jinju, 52828 Republic of Korea

**Keywords:** 3,4-methylenedioxy cinnamic acid, Hydratase-lyase, *Piper nigrium*, Piperonal, Piperonal synthase

## Abstract

Piperonal is a simple aromatic aldehyde compound with a characteristic cherry-like aroma and has been widely used in the flavor and fragrance industries. Despite piperonal being an important aroma in black pepper (*Piper nigrum*), its biosynthesis remains unknown. In this study, the bioinformatic analysis of the *P. nigrum* transcriptome identified a novel hydratase-lyase, displaying 72% amino acid identity with vanillin synthase, a member of the cysteine proteinase family. In in vivo substrate-feeding and in vitro enzyme assays, the hydratase-lyase catalyzed a side-chain cleavage of 3,4-methylenedioxycinnamic acid (3,4-MDCA) to produce 3,4-methylenedioxybenzaldehyde (piperonal) and thus was named piperonal synthase (PnPNS). The optimal pH for PnPNS activity was 7.0, and showed a *K*_m_ of 317.2 μM and a *k*_cat_ of 2.7 s^−1^. The enzyme was most highly expressed in the leaves, followed by the fruit. This characterization allows for the implementation of PnPNS in various microbial platforms for the biological production of piperonal.

**Supplementary Information:**

The online version contains supplementary material available at 10.1186/s13765-022-00691-0.

## Introduction

Piperonal (3,4-methylenedioxybenzaldehyde), also known as heliotropin, is a compound that contributes to the general fragrance and flavor of black pepper [1]. Piperonal has been widely used in the flavor and aroma industries to exploit its vanillin- or cherry-like fragrance. It is also a precursor for several synthetic drugs such as tadalafil (Cialis^®^) [2]. Piperonal has the potential to be used as a therapeutical compound due to its diverse pharmaceutical activities, such as antitubercular, anticonvulsant, antidiabetic, anti-obesity, and antimicrobial activities [3]. For example, piperonal was reported to prevent the accumulation of hepatic lipids and to upregulate insulin signaling molecules in mice under a high-fat diet to deter the occurrence of hyperlipidemia syndrome [4, 5].

Piperonal can be chemically synthesized to meet industrial demand with the following method: partial photocatalytic oxidation of piperonyl alcohol [6] and the chemical cleavage of piperine (or piperic acid) [7]. It is also supplied from different plant species such as vanilla, dill, and black pepper [3]. In black pepper, piperonal accumulates in the peppercorns [8]. Despite its wide uses, piperonal biosynthesis in pepper remains to be elucidated.

**Fig. 1:**
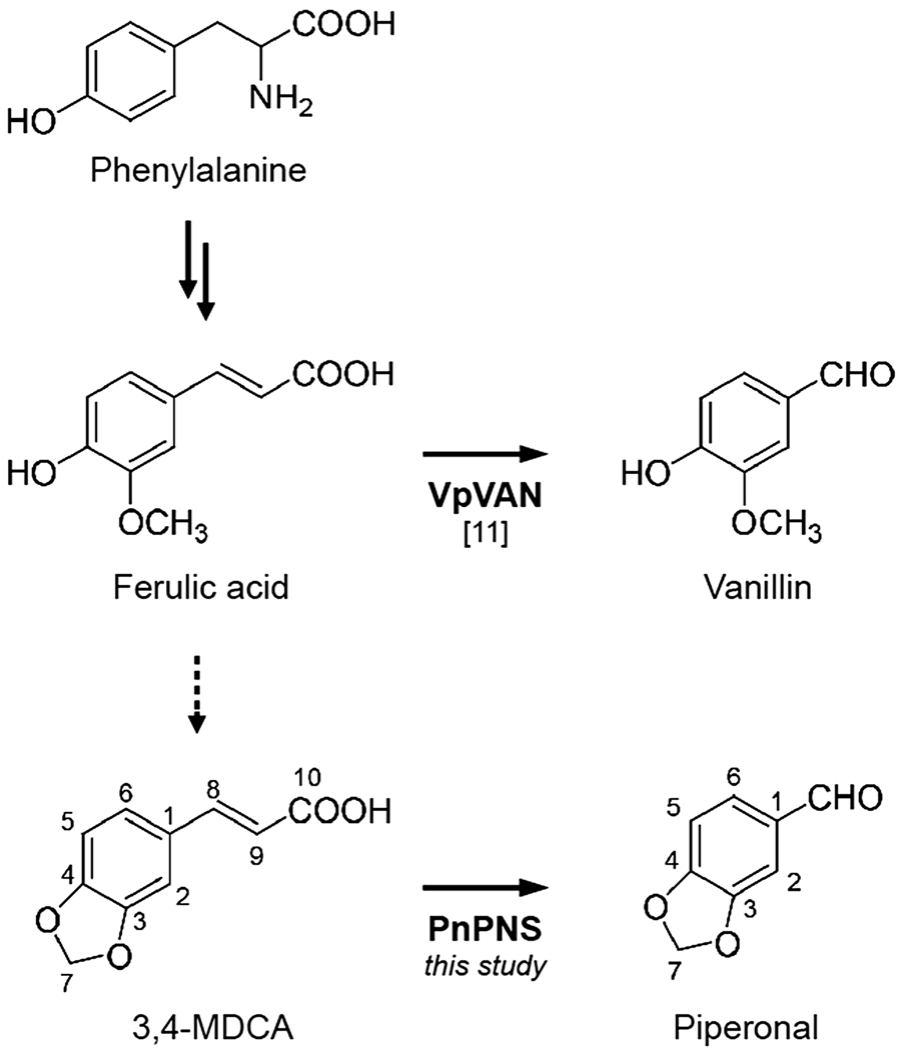
A proposed biosynthetic pathway of of piperonal from 3,4-MDCA. It has been postulated that phenylalanine is converetd to 3,4-methylenedioxycinnamic acid (3,4-MDCA). The side chain of 3,4-MDCA was cleaved by a *Piper nigrum* hydratase/lyase (PnPNS) to generate piperonal. *Vanilla planifolia* vanillin synthase (VpVAN) converts ferulic acid to vanillin. The solid arrows denote the catalytic steps with a known mechanism, the dashed arrow denotes a proposed reaction

Piperonal structurally resembles vanillin, where the 4-hydroxy-3-methoxy group replaces the 3,4-methylenedioxy moiety of piperonal (Fig. 1). Several microorganisms are known to produce vanillin from various substrates, including eugenol, ferulic acid, and curcumin [9]. Among the substrates, ferulic acid can be utilized by *Pseudomonas fluorescens* to produce vanillin in a CoA thioester-dependent biosynthetic reaction [10]. In this bacteria, hydroxycinnamate-CoA ligase-synthetase (HCLS) converts ferulic acid into feruloyl-CoA prior to the cleavage of the C–C double bond by hydroxycinnamoyl-CoA hydratase-lyase (HCHL). The HCHL reaction is thought to proceed in two steps, the hydration of the side-chain double bond of feruloyl-CoA and cleavage between the first and second carbon via a retro-aldol reaction to yield vanillin [10]. In contrast to HCHL in *P. fluorescens*, vanillin biosynthesis in *Vanilla planifolia* is the result of the shortening of ferulic acid`s side chain with a CoA thioester-independent hydratase-lyase reaction [11]. *V. planifolia* vanillin synthase (VpVAN) can accept ferulic acid and its glucoside to produce vanillin and vanillin glucoside, respectively, by splitting off the two-carbon unit [11].

The phenylpropanoid pathway suggests that piperonal is biosynthesized from phenylalanine via ferulic acid [12]. Recently, *P. nigrum* CYP719A37 was reported to produce piperic acid from 5-(4-hydroxy-3-methoxyphenyl)-2,4-pentadienoic acid by bridging the 4-hydroxy and 3-methoxy groups [13]. Similar P450s are shown in sesamin and canadine biosynthesis [14, 15]. In the present study, we identified a VpVAN-like hydratase-lyase gene encoding *P. nigrum* piperonal synthase. The enzyme can synthesize piperonal from an intermediate of the phenylpropanoid pathway, 3,4-MDCA, by a side-chain cleavage.

### Materials and methods

Materials and methods were described in Additional information. The primers used in this study were listed in Additional file 1: Table S1.

### Results and discussion

#### Isolation of a novel PnMCHL from P. nigrum

VpVAN, a hydratase-lyase belonging to the cysteine proteinase superfamily, was reported to catalyze the conversion of ferulic acid to vanillin in *Vanilla planifolia* (Fig. 1) [11]. We hypothesized that piperonal is biosynthesized by a homologous enzyme in pepper as ferulic acid and 3,4-MDCA share a similar structure. (Fig. 1). To test this hypothesis, the black pepper transcriptome was screened for homologues of VpVAN, and a full-length cDNA clone displaying 72% sequence identity with VpVAN, at the protein level, was identified (Additional file 1: Figure S1). This clone was named 3,4-methylenedioxycinnamic acid hydratase-lyase (PnMCHL).

PnMCHL contained six residues (Q156, C162, N301, N322, S323, and W324) known to form an active site, and six cysteines (C159-C202, C193-C235, and C293-C343) involved in conserved disulfide bridges in the cysteine proteinase family (Additional file 1: Figure S1) [11, 16]. On the basis of the conserved residues and high homology to VpVAN, we postulated that PnMCHL is likely to convert ferulic acid-like compounds to their respective aldehyde forms.

#### Functional assessment of PnMCHL

Before investigating the catalytic activity of PnMCHL in yeast, we tested the utilization and stability of its putative substrate in yeast. After feeding 3,4-MDCA to yeast cultures, the metabolites were analyzed by GC–MS. In the GC profile, decarboxylated 3,4-MDCA was detected (Additional file 1: Figure S2). The decaboxylation was most likely casued by two yeast enzymes, phenylacrylate decarboxylase (PAD1) and ferulate decarboxylase (FDC1), known to catalyze decarboxylations of various phenylpropenic acids in yeast [17]. To prevent the decarboxylation of 3,4-MDCA in yeast, we established a mutant yeast strain (YPH499 *ΔPAD1 ΔFDC1*) by the double disruption of *PAD1* and *FDC1* (Additional file 1: Figure S3). When 3,4-MDCA was fed to the mutant yeast strain, the decarboxylated product disappeared, indicating that the double-knockout mutant is unable to catabolize 3,4-MDCA (Additional file 1: Figure S2).

**Fig. 2:**
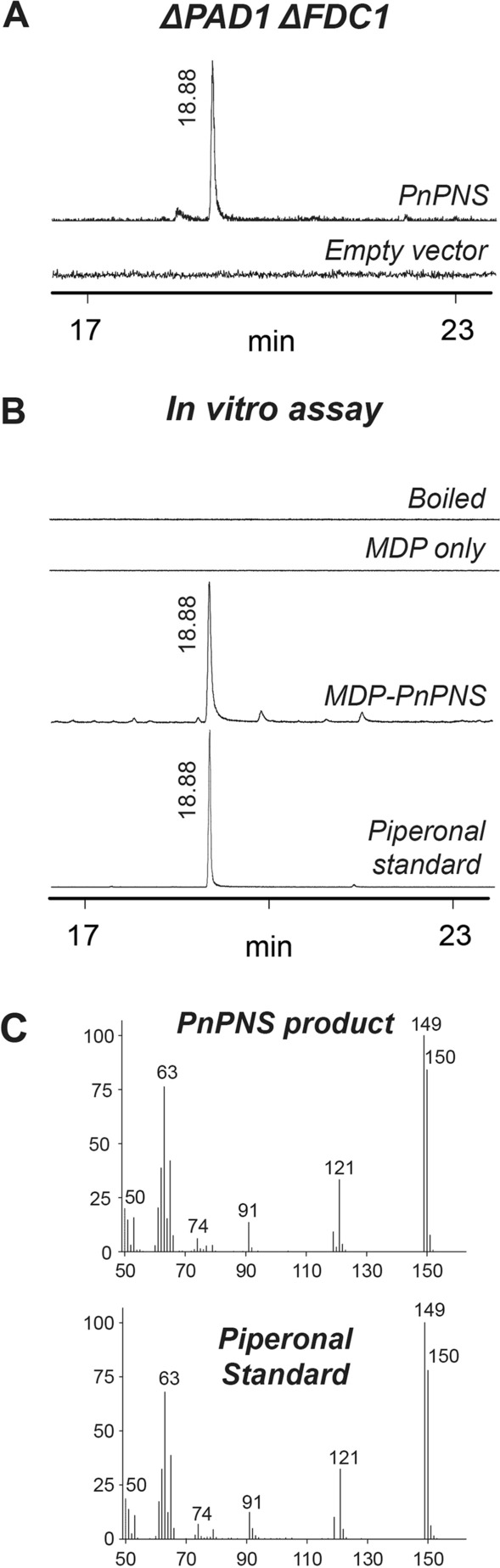
GC–MS chromatograms of PnPNS product. **A** GC–MS analysis of the culture extracts from empty-vector yeast and *PnPNS*-expressing yeast (YPH499 *ΔPAD1 ΔFDC1*). Extracted ion chromatograms at *m/z* 150 are shown. **B** In vitro recombinant PnPNS assays with 3,4-MDCA. Boiled, Boiled recombinant PnPNS; MDP only, maltose binding protein. **C** Mass spectra of the synthesized authentic standard and PnPNS product

In order to determine the catalytic activity of PnMCHL, the full length *PnMCHL* was expressed under the *Gal1* promoter in the pESC-Leu2d plasmid in YPH499 *ΔPAD1 ΔFDC1*. After feeding 3,4-MDCA to the yeast expressing *PnMCHL*, the metabolites were extracted using methylene chloride and analyzed by GC–MS. As a result, a new peak (*m/z* = 150) was detected from the methylene chloride extract, while no peak appeared from the empty vector control (Fig. 2A). A piperonal standard was chemically synthesized from 3,4-MDCA (Additional file 1: Figure S4), and its structure was fully elucidated by NMR analysis (Additional file 1: Figure S5). The new peak`s retention time and mass fragmentation were identical to those of the synthetic piperonal standard (Fig. 2B, C).

Functional characterization of PnMCHL was further performed using its recombinant enzyme. As cysteine proteinases localize to the endoplasmic reticulum (ER), the N-terminal 25 amino acids of PnMCHL were predicted to include ER-targeting sequences (Additional file 1: Figure S1). To properly express *PnMCHL* in *E. coli*, the first 25 amino acids of PnMCHL were truncated, and a maltose-binding protein (MBP) was tagged to the N-terminus. The maltose fusion enzyme was expressed in *E. coli* and purified through an MBP affinity column (Additional file 1: Figure S6). The purified PnMCHL recombinant enzyme (MBP-fused to the truncated PnMCHL) was incubated with 3,4-MDCA. In the GC–MS analysis, the same peak for piperonal was detected after feeding 3,4-MDCA (Fig. 2B). In contrast, the boiled and MDP only proteins could not produce piperonal. On the basis of this reult, we concluded that PnMCHL is able to catalyse the carbon double-bond cleavage of 3,4-MDCA to produce piperonal and, therfore, it was named piperonal synthase (PnPNS). Although PnPNS is similar to VpVAN, PnPNS could not convert ferulic acid to vanillin (Additional file 1: Figure S7).

A CoA-dependent catalytic reaction for vanillin biosynthesis has been reported in *Pseudomonas fluorescens* [9, 10]. This catalysis is comprised of two reactions. First, hydroxycinnamate-CoA ligase-synthetase (HCLS) catalyzes the formation of feruloyl-CoA from ferulic acid using ATP. Then, 4-hydroxycinnamoyl-CoA hydratase-lyase (HCHL) converts the feruloyl-CoA to vanillin and acetyl-CoA using NAD^+^ as a cofactor [9, 10]. In comparison PnPNS converts 3,4-MDCA to piperonal in the absence of ATP, CoA-SH, or NAD^ +^ in our in vitro assay. This indicates that PnPNS uses a CoA-independent mechanism.

On the other hand, the catalytic mechanism of cysteine proteinase is initiated from the oxyanion transition state [9, 11]. The oxyanion intermediate is hydrated and a subsequent retro-aldol elimination reaction cleaves the C–C bond. The oxyanion hole of VpVAN stabilizes the transition state of ferulic acid using hydrogen bonds from two residues (C162 and Q156, Additional file 1: Figure S1) [9, 11]. These two residues were also found in PnPNS [11]. Therefore, the PnPNS mechanism in black pepper is similar to VpVAN. The conversion of 3,4-MDCA might sequentially occur by two partial reactions, an initial hydration addition followed by a retro-aldol elimination reaction. The first reaction is initiated by the addition of a water molecule to the α and β-carbon linked, double-bond forming β-hydroxyl 3,4-MDCA. The second reaction undergoes a well-known retro-aldol elimination reaction, which results in the formation of piperonal and acetic acid (Additional file 1: Figure S4).

#### PnPNS enzyme characterization

**Fig. 3:**
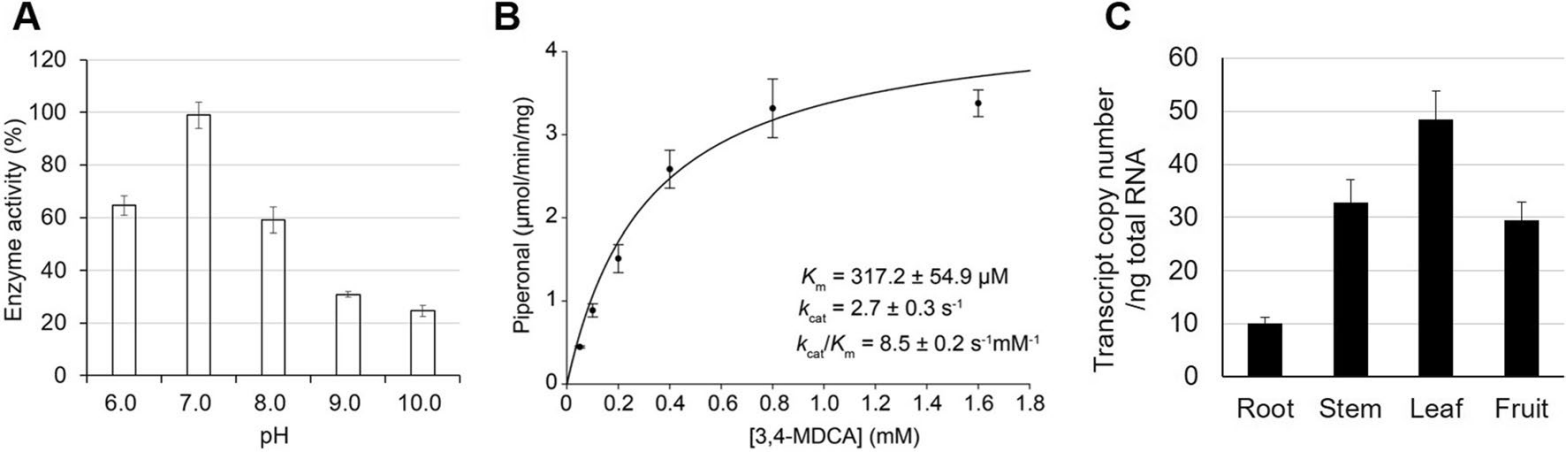
Characterizaion of PnPNS. **A** The optimal pH conditions for recombinant MBP-PnPNS. **B** Kinetic plot of recombinant PnPNS (mean ± S.D.; n = 3). The kinetic properties were calculated with the Michaelis–Menten equation using Sigma plot 12.0. **C** Transcript copy number of *PnPNS* from various tissues. The copy numbers were obtained from five biological replicates with four technical replicates

The optimal pH for PnPNS activity was investigated in the pH range between 6 to10. PnPNS showed the highest activity at pH 7, while 60% activity remained in pH 6 and pH 8. (Fig. 3A). To determine its kinetic protperties, purified recombinant PnPNS was incubated with 3,4-MDCA ranging from 50 µM to 1.6 mM, followed by GC–MS quantitation. The kinetic properties of PnPNS were determined to be *K*_*m*_ of 317.2 μM for 3,4-MDCA, *k*_*cat*_ of 2.7 s^−1^, which results in a catalytic efficiency (*k*_*cat*_/*K*_*m*_) of 8.5 s^−1^ mM^−1^ (Fig. 3B).

#### Expression of PnPNS in black pepper

Metabolite-profiling of the piper genus showed that piperonal and its derivatives are abundant in leaves and fruits [18]. Thus, we predicted the expression of *PnPNS* to be greatest in the black pepper leaves and fruits. To measure expression of *PnPNS* in black pepper, qRT-PCR was performed on root, stem, leaf and fruit tissue. PnPNS transcripts could be detcted in all four tissues examined, but leaves showed the highest expression (~ 5-fold higher expression in leaves than in roots) (Fig. 3C).

## Supplementary Information

**Additional file 1:** **Figure. S1** Alignment of PnPNS and VpVAN. **Figure. S2** GC-MS chromatograms of metabolites extracted from yeast fed with 3,4-MDCA. **Figure. S3** Generation of *ΔPAD1 ΔFDC1* yeast strain (YPH499 *ΔPAD1 ΔFDC1*). **Figure. S4** Piperonal synthesis by chemical and enzymatic reactions. **Figure .S5**^1^H-NMR spectrum of chemically synthesized piperonal. **Figure .S6** SDS-page gel image for purified recombinant PnPNS. **Figure. S7** In vitro PnPNS activity with ferulic acid. **Table S1.** List of primers used in this research. Under line indicated restriction enzyme site.

## Data Availability

Not applicable.
