# Supplementary material for: Piperonal synthase from black pepper (Piper nigrum) synthesizes a phenolic aroma compound, piperonal, as a CoA-independent catalysis
Source: Appl Biol Chem. 2022 Mar 24;65(1):20. doi: 10.1186/s13765-022-00691-0 (PMC8948145; doi:10.1186/s13765-022-00691-0)
Supplement: Supplementary file 1 — Additional file 1: Figure. S1 Alignment of PnPNS and VpVAN. Figure. S2 GC-MS chromatograms of metabolites extracted from yeast fed with 3,4-MDCA. Figure. S3 Generation of ΔPAD1 ΔFDC1 yeast strain (YPH499 ΔPAD1 ΔFDC1). Figure. S4 Piperonal synthesis by chemical and enzymatic reactions. Figure .S51H-NMR spectrum of chemically synthesized piperonal. Figure .S6 SDS-page gel image for purified recombinant PnPNS. Figure. S7 In vitro PnPNS activity with ferulic acid. Table S1. List of primers used in this research. Under line indicated restriction enzyme site. [file 13765_2022_691_MOESM1_ESM.docx]

Additional file Material

1 75

PnPNS (1) MASRLTLIPLFVVILAAAAAGSLSDEENPIRLVTDKAREAESAIHRTLGAAHHVMAFARFARRFGKQYSSVDEIR

VpVAN (1) MAAKLLFFLLFLVSALSV-ALAGFEEDNPIRSVTQRPDSIEPAILGVLGSCRHAFHFARFARRYGKSYGSEEEIK

76 150

PnPNS (76) KRFDIFVENLELIHSTNKRGLSYKLGINKFADLSWEEFKAHHLGAAQNCSATR-GTHKLTQAILPETKDWREEGI

VpVAN (75) KRFGIFVENLAFIRSTNRKDLSYTLGINQFADLTWEEFRTNRLGAAQNCSATAHGNHRFVDGVLPVTRDWREQGI

151 225

PnPNS (150) VSPVKNQGHCGSCWTFSTTGALEAAYTQATGKSISLSEQQLVDCASGFNNFGCNGGLPSQAFEYIKYNGGLDTEE

VpVAN (150) VSPVKDQGSCGSCWTFSTTGALEAAYTQLTGKSTSLSEQQLVDCASAFNNFGCNGGLPSQAFEYVKYNGGIDTEQ

226 300

PnPNS (225) SYPYAGVNGICGYKIENIGVKVAESVNITEGAEDELKHAVALVRPVSIAFQVVHDFRSYKGGVYTSQECGSAPMD

VpVAN (225) TYPYLGVNGICNFKQENVGVKVIDSINITLGAEDELKHAVGLVRPVSVAFEVVKGFNLYKKGVYSSDTCGRDPMD

301 357

PnPNS (300) VNHAVLAVGYGVENGVPYWLVKNSWGNDWGVDGYFKIELGKNM**C**GVATCASYPILSL

VpVAN (300) VNHAVLAVGYGVEDGIPYWLIKNSWGTNWGDNGYFKMELGKNMCGVATCASYPIVAV

**Additional file 1 Figure S1. Alignment of PnPNS and VpVAN.**

PnPNS was aligned to VpVAN (accession number AKG47593). The characteristic conserved residues of cysteine proteinases are present in PnPNS. Red underline, a putative endoplasmic reticulum (ER) targeting sequence; Six blue residues, components of the active site in cysteine proteinases; Residues with red-backgrounds, conserved cysteines forming disulfide bridges in cysteine proteinases.; Residues with yellow backgrounds, residues involved in forming an oxyanion hole for stabilizing the transition state of ferulic acid.


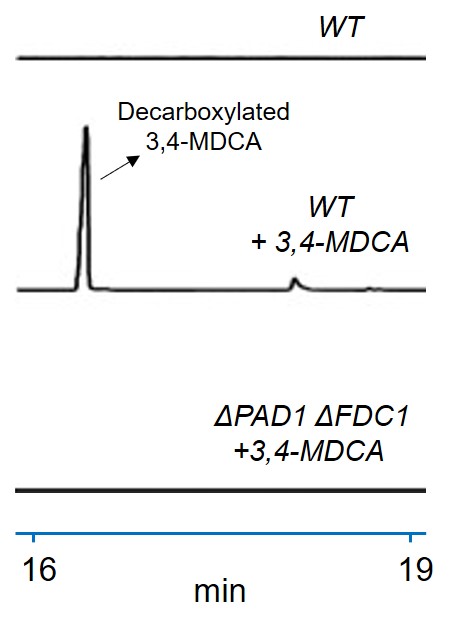


**Additional file 1 Figure S2. GC-MS chromatograms of metabolites extracted from yeast fed with 3,4-MDCA**

3,4-MDCA was fed to *Saccharomyces cerevisiae* strain YPH499 (WT) and *ΔPAD1 ΔFDC1* mutant strains. The metabolites were extracted with methylene chloride, and analyzed by GC-MS.

**
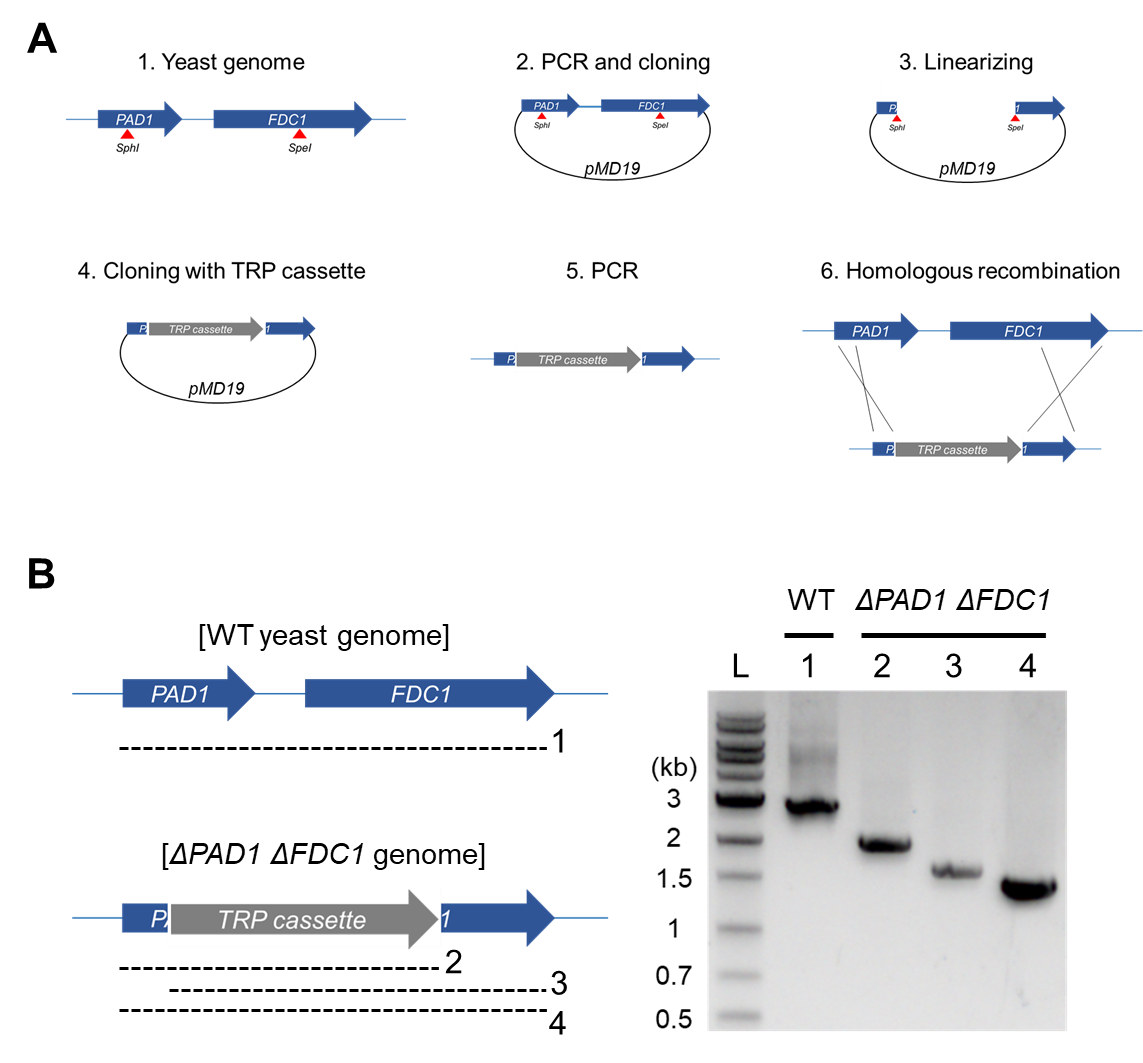
**

**Additional file 1 Figure S3. Generation of *ΔPAD1 ΔFDC1* yeast strain (YPH499 *ΔPAD1 ΔFDC1*).**

**A**. Strategy of yeast *PAD1* and *FDC1* disruption by *TRP cassette* insertion. Tryptophan synthase expression cassette was inserted between *PAD1* and *FDC1* by homologous recombination to inactivate PAD1 and FDC1. **B**. Confirmation of the inserted gene by PCR. The dashed lines represent PCR products used for the confirmation.

**Additional file 1 Figure S4. Piperonal synthesis by chemical and enzymatic reactions.**


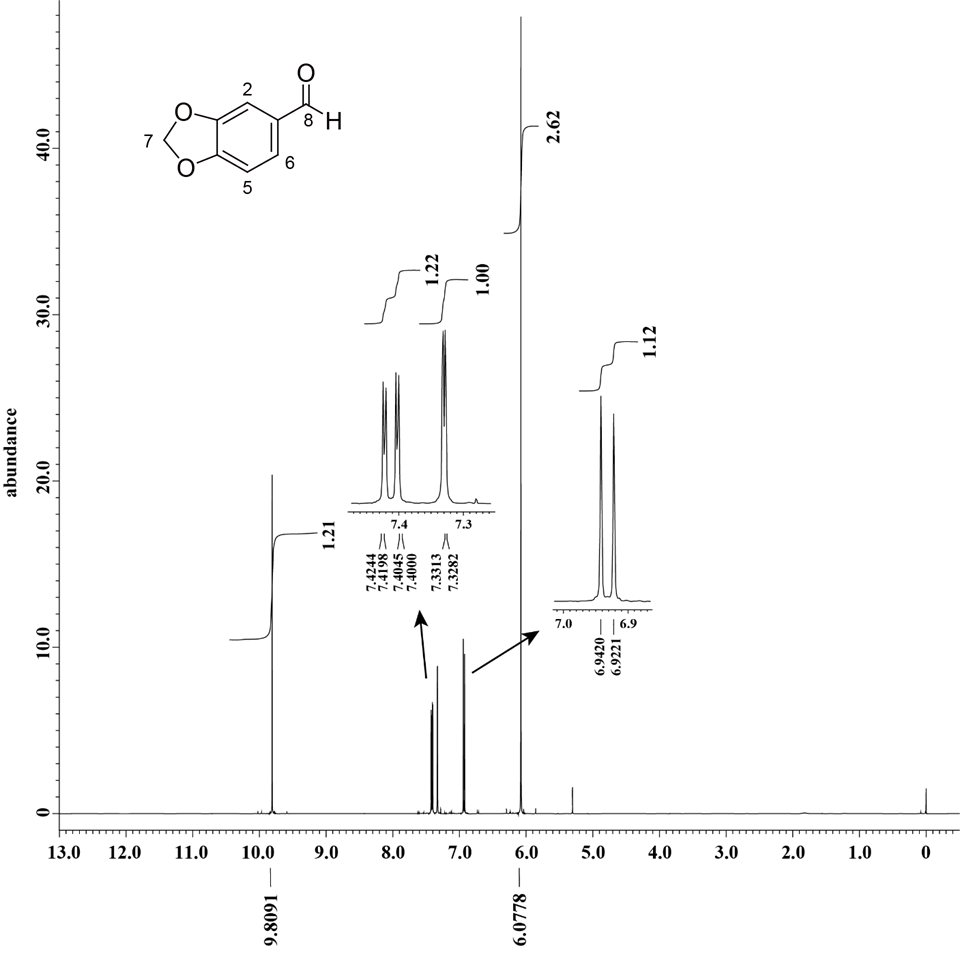


**Additional file 1 Figure S5. ^1^H-NMR spectrum of chemically synthesized piperonal**


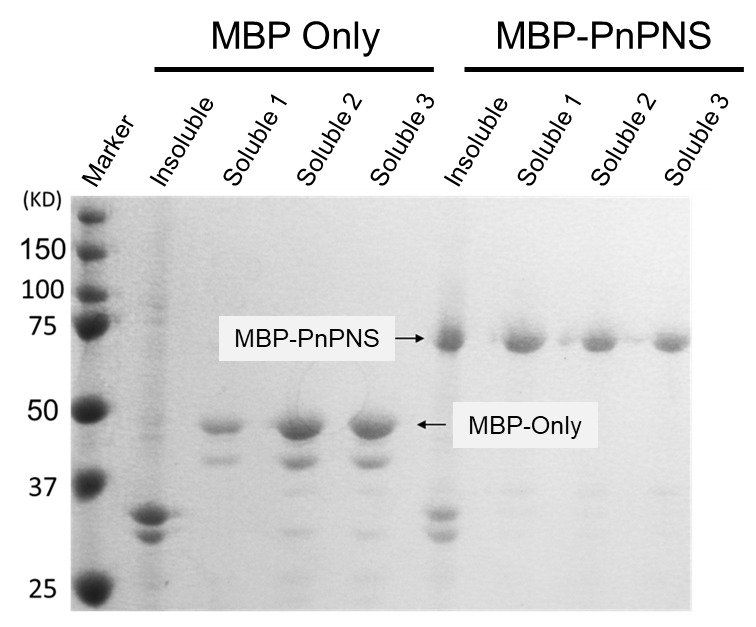


**Additional file 1 Figure S6. SDS-page gel image for purified recombinant PnPNS.**

A maltose binding protein (MBP) was tagged to the N-terminus of PnPNS, and the MBP-PnPNS was expressed in *E. coli*. MBP was also expressed in *E. coli* as a negative control. After cell lysis, the soluble fraction was bound to amylose resin and eluted using 10 mM maltose.


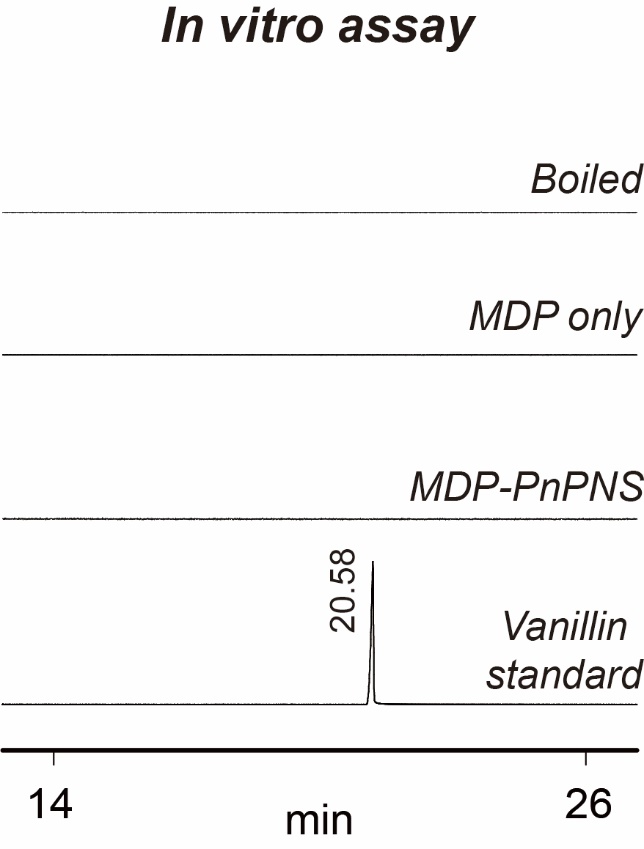


**Additional file 1 Figure S7. In vitro PnPNS activity with ferulic acid.**

Recombinant PnPNS was incubated with fereulic acid. The product was extracted with methylene chloride, and analyzed by GC-MS. Boiled, boiled recombinant PnPNS; MDP only, Maltose binding protein.

**Additional file result**

***Chemical preparation of piperonal standard***

Piperonal was chemically synthesized from 3,4-MDCA, which served as a standard (Fig 2; Additional file 1 Fig. 4). The chemical degradation of 3,4-MDCA proceeded in the oxidant NaOH and reductant CuSO_4_·5H_2_O at a boiling temperature. The combined oxidizing agent of OH^-^ and reducing agent of Cu^2+^ ion prevented the oxidation of piperonal (C6C1) to piperonyl acid. The production of piperonal was also confirmed by ^1^H NMR spectroscopy (Additional file 1 Fig. 5). The chemical shifts were 6.08 (s, 2H, H_7_), 6.93 (d, *J* =8.2, 1H, H_5_), 7.33 (d, *J*=1.5, 1H, H_2_), 7.42 (dd, *J*=8.2, 1.6, 1H, H_6_) and 9.81 (s, 1H, H_8_) ppm. The mass of the synthesized piperonal was confirmed to be 150 by GC-MS analysis (M^+^ ion with *m/z* 150, Fig. 2)*.*

**Materials and Methods**

***Plant material and RNA isolation***

Immature peppercorns of *P. nigrum* seeds and other tissue samples of root, stem and leaves were obtained from the Prince of Songkla University garden in Thailand (Gift from Prof. Juraithip Wungsintaweekul). Total RNA was isolated through the CTAB method [1, 2]. Subsequently, cDNA was synthesized (PhileKorea, Korea) from 2 μg of purified total RNA by reverse-transcription using an oligo (dT_17_) primer with the following PCR condition: 45 min for 42℃, 10 min for 70℃ to denature RTase activity, finally held at 16℃.

***Preparation of piperonal by chemical reaction***

Piperonal was prepared from 3,4-MDCA using a previously described procedure [3] with slight modification. Crude piperonal was extracted from the reaction mixture 3 times with 5 ml of methylene chloride followed by concentration to 1 ml under gentle nitrogen flow. The crude piperonal was purified by open column chromatography on a silica gel glass column (10×1 cm; 60 Å, 40-63 μm particle size, Merck). The column was pre-washed with 30 ml of methylene chloride:hexane (4:1), 30 ml methylene chloride, and finally 30 ml of methylene chloride:ethyl acetate (9:1). The concentrated methylene chloride fraction containing the reaction product was analyzed with GC-MS (6890 GC/ 5973 mass detector, Agilent, U.S.A.) and ^1^H-NMR spectrometer (JNM-LA400, JEOL, Japan).

***Isolation of PnMCHL***

Using the previously identified *VpVAN* as a query [4], homologous gene candidates were searched for in the transcriptome database of fresh black pepper fruit (tBLASTn, BioEdit version 7.2). Based on the sequence of the *PnMCHL* candidate, the full length ORF was amplified from cDNA using primer set 1/2 (Additional file 1 Table 1). The PCR product was cloned into the *pMD19* T-vector (Takara, Japan).

***Yeast gene disruption***

To disrupt *PAD1* (phenylacrylic acid decarboxylase, accession No. *YDR538W*) and *FDC1* (ferulic acid decarboxylase, accession No. *YDR539W*) in *Saccharomyces cerevisiae* strain YPH499 (Agilent, U.S.A.), *PAD1* and *FDC1* genes (total 2.7kbp) were amplified using primer set 3/4 (Additional file 1 Table 1) and the purified PCR product was ligated into the *pMD19* T-vector (Additional file 1 Fig. 3). The vector was then digested with *SphI* and *SpeI*. Then the TRP cassette (Tryptophan synthase gene expression cassette, 1.1kbp) was amplified with primer set 5/6 from the *pESC-TRP* vector and was ligated into the linearized vector (Additional file 1 Fig. 3). The *PAD1*-TRP cassette-*FDC1* was amplified by primer set 3/4, and 5 μg of PCR product was transformed into yeast strain YPH499 using the lithium acetate method [5]. The *pad1-fdc1* knock-out yeast was selected with SC-Trp, and named YPH499 *ΔPAD1 ΔFDC1*

***Heterologous expression in mutated yeast***

For the functional expression of the *PnMCHL* candidate in yeast*,* the *PnMCHL* ORF was amplified with primer set 7/8 and ligated into *pESC*-LEU2d at the *Bam*HI and *Nhe*I sites. The cloned vector was then transformed to YPH499 *ΔPAD1 ΔFDC1*. A transformed single colony on the SC-Leu-Trp plate was cultured in 3 ml of liquid medium for 16 h. The cells were inoculated into 30 ml of SC-Leu/-Trp medium with 2% galactose, 0.2% glucose in a 150 mL flask. After cultivating yeast for 3 days at 30℃ at 200 rpm, 0.2 mM of 3,4-MDCA was added into the culture. After incubation, the medium was directly extracted with 3 ml of methylene chloride for GC-MS analysis.

***Preparation of recombinant PnPNS***

To prepare the recombinant PnPNS, the putative N-terminal ER (endoplasmic reticulum) transit peptide (75 nucleotides) was truncated from *PnPNS* using primer set 9/10. The PCR product was cloned into *pET*-*MBP* at the *Nde*I and *Xho*I sites to add an N-terminal MBP tag using the Gibson Assembly^®^ Cloning Kit (NEB, U.K.). The *pET-MBP-PnPNS* vector was then transformed into Rosetta™ 2(DE3) (Agilent, U.S.A.). A single colony was inoculated into 5 ml LB (Luria-Bertani) broth (MBcell, U.S.A.) with 50 μg/ml kanamycin, and the cells were cultivated at 37℃ overnight. The seed culture was inoculated (1/100, v/v) into 50 ml of fresh LB broth with the same concentration of antibiotics. Incubation at 37℃ continued until the OD_600_ reached 0.5, at which point 0.1 mM IPTG was added to the culture and the incubation temperature was reduced to 18℃ to induce protein expression. The culture was further incubated for 16 h and the cells were collected by centrifugation at 4000 *g* for 5 min at 4℃. The cell pellet was resuspended in 5 ml of lysis buffer (50 mM potassium phosphate buffer, pH 7.0). The suspension was then sonicated (Sonic Dismembrator 550, Fisher Scientific, U.S.A.) for a total of 5 min with repeated 3 s pulses on and 1 s pulses off. The soluble protein fraction was collected by centrifugation at 6000 *g* for 30 min at 4℃. The supernatant was applied to an Amylose Resin (NEB, U.K.) column and the MBP tagged enzyme was eluted with a lysis buffer containing 10 mM maltose.

***In-vitro assay and enzyme kinetics***

To investigate the optimal pH conditions, the *in vitro* enzyme assay was performed at pH 6, 7, 8, 9 and 10. For the kinetics study, 26.7 nM of purified recombinant PnPNS was mixed with 0.05 to 1.6 mM 3,4-MDCA serially diluted into six different concentrations in 500 μl of reaction buffer (100 mM phosphate buffer, pH 7.0, 10mM Mg^2+^ and 2.5 mM DTT). The mixture was then incubated for 15 min at 30℃, and the reaction was stopped by boiling for 3 min. All assays were performed in triplicate and the piperonal peak-area was determined in the extracted-ion chromatogram (EIC) mode by GC-MS. The six point serial dilutions of authentic piperonal (10, 20, 40, 60, 80 and 100 nM) were used for a standard curve. The kinetic parameters of recombinant PnPNS with 3,4-MDCA was calculated on SigmaPlot 12.0 with the Michaelis-Menten equation (*ν*=*V_max_*/(1*+K_m_*/[*S*])).

***GC-MS analysis***

Gas chromatography (Agilent model 6890, U.S.A.) equipped with a mass spectrometer was used to analyze the terpene products under the following conditions: helium gas at 1 ml/min, 1 μl injection at 220℃, Zebron ZB-5MSi capillary column (60 m × 0.25 mm × 0.25 μm), a temperature program from 70℃ to 250℃ at 5℃/min and then at 20℃/min to 320℃ (2 min hold). The coupled mass spectrometer (Agilent HP 5973, U.S.A.) used the following parameters: transfer line temperature set at 320℃, source temperature at 260℃, quadrupole temperature at 150℃ and ionization potential at 70 eV. Scan range was 50 to 200 *m/z* units.

***Transcription level analysis by qRT-PCR***

Transcription levels of *PnPNS* were determined by qRT-PCR according to the manufacturer`s protocol (QuantiMix SYBR Kit PCR system, PhileKorea, Korea) with Rotor-Gene 2000 Real Time Cycler (Corbett Research, Australia) using primer set 11/12 (Additional file 1 Table 1). A standard curve was generated based on the Ct value of serial dilutions of a *PnPNS* containing plasmids ranging from 10^2^ to 10^7^ copys/μl. The copy numbers were calculated as described [6]. Each data point was obtained from quintuple biological samples each composed of quadruple technical replicates.

**Reference**

1. Chang S, Puryear J, Cairney J (1993) A simple and efficient method for isolating RNA from pine trees. Plant Mol Biol Rep 11:113-116

2. Sambrook J, Russell DW (2006) Purification of nucleic acids by extraction with phenol: chloroform. Cold Spring Harb Protoc prot4455

3. Pearl IA (1950) Method of synthesizing syringaldehyde. U.S. Patent 2,516,412[P]. 1950-7-25

4. Gallage NJ, Hansen EH, Kannangara R, Olsen CE, Motawia MS, Jørgensen K, Holme I, Hebelstrup K, Grisoni M and Møller BL (2014) Vanillin formation from ferulic acid in *Vanilla planifolia* is catalysed by a single enzyme. Nat Commun 5:4037 doi: 10.1038/ncomms5037

5. Gietz RD, Schiestl RH (2007) High-efficiency yeast transformation using the LiAc/SS carrier DNA/PEG method. Nat Protoc 2:31-34

6. Yin JL, Shackel NA, Zekry A, McGuinness PH, Richards C, Van Der Putten K, McCaughan GW, Eris JM and Bishop GA (2001) Real-time reverse transcriptase–polymerase chain reaction (RT–PCR) for measurement of cytokine and growth factor mRNA expression with fluorogenic probes or SYBR Green I. Immunol Cell Biol 79:213-221

**Additional file 1 Table S1. List of primers used in this research. Under line indicated restriction enzyme site.**

| **No.** | **Usage** | **Sequences (5’🡪3’)** |
| --- | --- | --- |
| 1 | Isolation of PnPNS | atggcgtctcgcctcactctc |
| 2 |  | ttacagagagagaataggataagatg |
| 3 | Disruption of PAD1 & FDC1 | atgctcctatttccaagaagaa |
| 4 |  | ttatttatatccgtaccttttcca |
| 5 |  | acatgcatgcaccataaacgacattactatatata |
| 6 |  | ggactagtaatttcctgatgcggtattttc |
| 7 | PnPNS expression in yeast | cgggatccgatggaggagaatccgatccg |
| 8 |  | acgcgtcgaccagagagagaataggataagatg |
| 9 | Recombinant PnPNS | cgccatatggaggagaatccgatccggctt |
| 10 |  | cccgctcgagcagagagagaataggata |
| 11 | qRT-PCR of PnPNS | cagcttacactcaagcaacggg |
| 12 |  | ggaagacctccattgcagcca |
